# Clinical Items for Geriatric Patients with Post-Stroke at Discharge or Transfer after Rehabilitation Therapy in a Chronic-Phase Hospital: A Retrospective Pilot Study

**DOI:** 10.3390/healthcare10081577

**Published:** 2022-08-19

**Authors:** Masatoshi Koumo, Akio Goda, Yoshinori Maki, Kouta Yokoyama, Tetsuya Yamamoto, Tsumugi Hosokawa, Ryota Ishibashi, Junichi Katsura, Ken Yanagibashi

**Affiliations:** 1 Department of Rehabilitation, Hikari Hospital, Otsu 520-0002, Japan; 2 Department of Physical Therapy, Faculty of Health Sciences, Kyoto Tachibana University, Kyoto 607-8175, Japan; 3 Department of Neurosurgery, Hikone Chuo Hospital, Hikone 522-0054, Japan; 4 Department of Neurosurgery, Kitano Hospital, Tazuke Kofukai Medical Research Institute, Osaka 530-0025, Japan

**Keywords:** chronic-care hospital, destination, functional independence measure, rehabilitation therapy, stroke

## Abstract

Clinical factors related to destination after rehabilitation therapy for geriatric patients with post-stroke in chronic-phase hospitals have not been elucidated. This study analyzed the clinical characteristics of geriatric patients with post-stroke at discharge/transfer after rehabilitation therapy in a chronic-phase hospital. Fifty-three patients (20 men, 33 women; mean age 81.36 ± 8.14 years) were recruited (the period analyzed: October 2013–March 2020). Clinical data were statistically analyzed among patients discharged to homes or facilities for older adults or transferred to another hospital. In addition, we analyzed the clinical items at discharge and transfer after rehabilitation therapy using a decision tree analysis. Twelve patients were discharged, eighteen were discharged to facilities for older adults, and twenty-three were transferred to another hospital. There were significant differences in the modified Rankin Scale, admission dates, functional independence measure (FIM) score, and Barthel Index score in the three groups (*p* < 0.05). Patients with motor subtotal functional independence scores of ≥14 (chronologically improved ≥5) after rehabilitation therapy for <291 days were more likely to be discharged home. Patients in a chronic-phase hospital who improved within a limited period were discharged to their homes, whereas those who were bedridden tended to be transferred to another hospital.

## 1. Introduction

A major concern in Japanese society is the increasing number of older adults requiring care for being bedridden [1]. Stroke is a major cause of a bedridden status for geriatric patients in Japan. In 2017, more than one million Japanese people underwent medical treatment for stroke [1]. Although improved medical treatment for stroke contributed to reducing the mortality related to stroke, it remains the second leading cause of bedridden status among patients and the third leading cause of death in Japan [1]. Therefore, the prevention of a stroke onset and recovery treatment after stroke events, such as rehabilitation therapy, is indispensable in the aging Japanese society. Rehabilitation therapy for post-stroke events is performed in three types of hospitals: acute, recovery, and chronic. Geriatric patients who can be independent or supported in their domestic environment can be discharged home after medical treatment, including rehabilitation therapy during the acute phase of stroke (usually within 2 months after a stroke onset). Meanwhile, patients who cannot be discharged from an acute-phase hospital often require transfer to a recovery or chronic-phase hospital [1].

Previous studies have reported clinical predictors of patients with post-stroke related to destinations (such as home, facilities for older adults, or another hospital) after rehabilitation therapy in acute- and recovery-phase hospitals [2,3,4,5,6,7,8,9]. However, to our knowledge, little is known about the clinical characteristics of geriatric patients with post-stroke at discharge or transfer after rehabilitation therapy in chronic-phase hospitals. Therefore, this study analyzed the clinical characteristics of geriatric patients with post-stroke upon discharge or transfer after rehabilitation therapy in a chronic-phase hospital. We aimed to clarify the clinical characteristics of geriatric patients with post-stroke at the termination of rehabilitation therapy in a chronic-phase hospital.

## 2. Materials and Methods

### 2.1. Study Design

This retrospective cohort study was approved by the ethics committee of Hikari Hospital (2 April 2021). Informed consent was obtained from all the participants.

We reviewed the medical records of Hikari Hospital between October 2013 and March 2020. The inclusion criteria were as follows: (1) aged 65 years or older, (2) admitted to the chronic-phase ward, (3) discharged by April 2020, (4) diagnosed with a stroke, and (5) receiving rehabilitation intervention. Candidates were excluded if they (1) died during admission or (2) if their clinical data were lacking.

### 2.2. Collection of Clinical Data 

We collected data on the following variables: sex (male/female), age (years), utilization of long-term care insurance (yes/no), the existence of housemates (yes/no), admission dates until discharge (days), initiation time of rehabilitation therapy from stroke onset (days), underlying stroke disease (infarction/intracranial hemorrhage), location of stroke lesion (supratentorial/infratentorial), laterality of stroke lesion (right/left), feeding upon admission (oral/non-oral), period of rehabilitation therapy during admission (days), destination after discharge (home/facilities for the older adults/transfer to another hospital), modified Rankin Scale (mRS) (on admission/at discharge), functional independence measure (FIM) score, Barthel Index (BI) score (at the time of initiating rehabilitation therapy/at discharge), and chronological change in FIM and BI scores.

### 2.3. FIM and BI

The FIM has two sections: motor subtotal (eating, grooming, bathing, upper body dressing, lower body dressing, toileting, bladder management, bowel management, bed/chair/wheelchair transfer, toilet transfer, tub/shower transfer, locomotion in the form of walking and/or wheelchair use, and stair use) and cognitive subtotal (comprehension, expression, social interaction, problem-solving, and memory) scores. Each item was scored from 1 to 7 according to the patient’s activities of daily living (ADLs). The minimum and maximum FIM scores were 18 and 126, respectively.

The BI consists of ten items: feeding, bathing, grooming, dressing, bowel control, bladder control, toilet use, transfer, mobility, and stair use. Each item was evaluated with scores of 0, 5, 10, or 15, according to the patient’s ability to perform daily activities. The minimum and maximum BI scores were 0 and 100, respectively.

### 2.4. Statistical Analysis

The data collected from patients discharged to their homes or to facilities for older adults and those transferred to another hospital were statistically analyzed using Kruskal–Wallis tests (post hoc analysis: Bonferroni correction), one-way analysis of variance (post hoc analysis: Bonferroni correction), and Fisher’s exact tests (post hoc analysis: Holm correction). A decision tree analysis was performed using all clinical data items to examine the differences among the clinical items of patients with post-stroke at discharge or transfer. IBM SPSS Statistics for Windows (version 24.0; IBM Corp., Armonk, NY, USA) was used for all statistical analyses. Statistical significance was set at *p* < 0.05.

## 3. Results

This study enrolled 53 patients (male-to-female ratio, 20:33). The mean age ± standard deviation was 81.36 ± 8.14 years. Twelve patients were discharged home, eighteen were discharged to facilities for older adults, and twenty-three were transferred to another hospital. The results of the three-group comparisons are shown in Table 1. The mRS scores upon admission and discharge were significantly higher in the other hospital group than in the other two groups (home and geriatric facilities) (*p* < 0.05). FIM (motor subtotal/cognitive subtotal/total) and BI scores upon admission and discharge were significantly lower in the other hospital group than in the other two groups (home and geriatric facilities) (*p* < 0.05). Chronological changes in the FIM and BI scores were significantly higher in the home group than in the facilities for the older group (*p* < 0.05). For the feeding items, the percentage of non-oral intake was significantly higher in the other hospital group than in the facilities for the geriatric group (*p* < 0.05). The admission dates were significantly longer in the facilities for the geriatric group than in the home group (*p* < 0.05). The transfer FIM score at discharge was significantly higher in the home group than in the other two groups (facilities for the geriatric and other hospitals) (*p* < 0.05).

### Decision Tree Analysis

**Figure 1:**
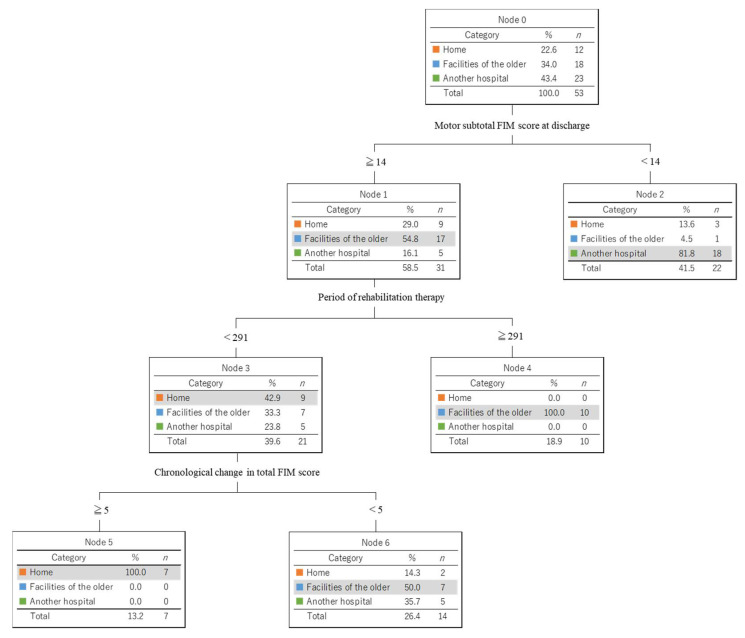
Results of decision tree analysis. FIM motor subtotal score, period of rehabilitation therapy, and chronological change of total FIM score were identified as discriminators at discharge or transfer after rehabilitation therapy.

A decision tree analysis identified the following discriminators: motor subtotal FIM score at discharge, rehabilitation therapy period, and chronological change in the total FIM score. The best discriminator was the motor subtotal FIM score (≧14 or <14). Patients with motor subtotal FIM scores of <14 were categorized for transfer to another hospital. In this group, three patients were discharged home, one patient was discharged to a facility for older adults, and eighteen patients were transferred to another hospital. Among the patients with scores ≥ 14, the next best discriminator was the rehabilitation therapy period (≥291 days or <291 days). Patients with a motor subtotal FIM score ≥ 14 who underwent rehabilitation therapy for at least 291 days were discharged to facilities for older adults. The third-best discriminator was the chronological change in the total FIM score (≥5 or <5). Seven patients with a chronological change in the total FIM score of ≥5 were discharged to their homes. The classification accuracy of the decision tree analysis was 79.2% (58.3% for patients discharged to home, 94.4% for those discharged to facilities for older adults, and 78.3% for those transferred to another hospital) (Figure 1).

## 4. Discussion

This study analyzed the clinical characteristics of geriatric patients with post-stroke at discharge and transfer after rehabilitation therapy at our chronic-care hospital by comparing three groups of patients: those discharged to their homes, those discharged to facilities for older adults, and those transferred to another hospital. Between the three groups, the ADLs evaluated using the FIM and BI scores, admission dates, and feeding status (oral/non-oral) were significantly different. In addition, the decision tree analysis results showed that patients with subtotal motor FIM scores at discharge ≥ 14, period of rehabilitation therapy < 291 days, and chronological change in total FIM score ≥ 5 were more likely to be discharged home. 

Patients undergoing rehabilitation therapy following stroke events are frequently evaluated using the FIM [2,10,11,12,13,14,15]. The correlations between the outcome of rehabilitation therapy after stroke events and FIM scores (motor subtotal/cognitive subtotal/total) in acute- and recovery-phase hospitals have been described [4,5,6,7,9,16,17]. The BI is also widely used to evaluate patient performance and to predict outcomes related to rehabilitation therapy [8,18,19]. In the present study, the FIM/BI scores at admission and discharge were also significant factors for discharge/transfer after rehabilitation therapy of geriatric post-stroke patients in a chronic-care hospital. Notably, the chronological change in the total FIM score was significant in the decision tree analysis. These results suggest that improvements in physical performance during rehabilitation therapy may result in a discharge home from a chronic-phase hospital. Previous reports identified FIM scores as predictors of discharge destination in geriatric stroke patients [6,20,21,22,23]. It has also been reported that improvements in the FIM score during hospitalization can lead to household discharge [24]. The results of the present study were consistent with the reports of these previous studies. Therefore, positive and effective rehabilitation therapy resulting in improved ADLs in geriatric stroke patients is warranted in chronic-care hospitals to increase the likelihood of being discharged home.

The results of the present study identified the period of rehabilitation therapy as the second discriminator of patient destination after rehabilitation therapy. Patients with total FIM scores of ≥14 and undergoing rehabilitation therapy for ≥291 days were discharged to facilities for older adults. A previous study [25] has also reported that prolonged length of hospital stay is strongly associated with discharge to a geriatric facility for patients with post-stroke sequelae. There are cases in which a discharge home from a chronic-care hospital becomes impossible despite the patient’s ability to perform ADLs due to factors such as the caregivers and the home environment. Compared with patients discharged home or transferred to another hospital, patients discharged to facilities for older adults had to wait because of the limited number of rooms at facilities for older adults. Consequently, the patients continued to be admitted and underwent rehabilitation therapy. The discharge of stroke patients to geriatric care facilities is a bottleneck in discharge coordination and is likely to prolong the length of discharge [26]. Therefore, it is useful to pay attention to the prolonged duration of rehabilitation treatment (length of stay) to predict the discharge transition of geriatric stroke patients admitted to chronic-care hospitals.

In this study, a significantly higher percentage of patients transferred to another hospital were parenteral, confirming that feeding status was also important. This may be because the administration of gastrostomy, tubal feeding, and central venous feeding can be complicated for staff working in facilities for older adults [27,28]. Furthermore, the mRS results at admission and discharge showed that patients transferred to another hospital had significantly more severe diseases. Previous studies [29,30] have also reported that the severity of illness, as assessed by the mRS during hospitalization, affects poor discharge outcomes (discharge to another location other than home). Therefore, feeding status and disease severity should be confirmed when predicting where geriatric stroke patients will be discharged from chronic-care hospitals.

### Limitations

This retrospective study was conducted at a single chronic-care hospital. The number of enrolled patients was limited, possibly because patients in chronic-care hospitals tend to be admitted longer than those in acute or recovery-care hospitals. Future multicenter studies should include a larger number of patients. As multiple rehabilitation therapists evaluated patients’ performance, subjective bias could not be completely excluded. In this regard, efforts should be made to minimize bias among evaluators by providing training in evaluation. In addition, this study could not examine the influence of modifiable stroke risk factors, and future studies should collect and analyze a wider range of data. In addition, we did not evaluate the types of hospitals to which the patients were transferred, such as acute-phase or other chronic-phase hospitals. Thus, whether patients were transferred to another hospital because of an improved (or at least stable) status or a sudden aggravated status remains unclear and should be addressed in future studies.

## 5. Conclusions

In this study, patients with post-stroke whose ADLs improved with rehabilitation therapy within a short period were more likely to be discharged to their homes. Meanwhile, a longer period of rehabilitation therapy and low motor subtotal FIM scores were related to discharge to facilities for older adults and transfer to another hospital. Rehabilitation therapy resulting in improved ADLs and early discharge also contributed to a discharge home in the chronic phase after stroke.

## Figures and Tables

**Table 1:** Comparison of the fundamental information and measurements among the three groups.

	Home (*n* = 12)	Geriatric Facilities (*n* = 18)	Another Hospital (*n* = 23)	*p*-Value	Post Hoc Analysis		
					Home—Geriatric Facilities	Home—Another Hospital	Geriatric Facilities—Another Hospital
Age * (years)	78.8 ± 9.1	81.5 ± 8.5	82.6 ± 7.4	0.419	1.000	0.5712	1.000
Sex ☨ (male/female)	(7/5)	(5/13)	(8/15)	0.222	0.408	0.565	0.741
Utilization of long-term care insurance ☨ (Yes/No)	(7/4)	(13/4)	(16/5)	0.703	1.000	1.000	1.000
Housemates ☨ (yes/no)	(10/2)	(10/8)	(18/5)	0.165	0.537	1.000	0.537
Feeding ☨ (oral/non-oral)	(9/3)	(18/0)	(7/16)	<0.001	0.059	0.059	<0.001
Underlying stroke ☨ (infarction/intracranial hemorrhage)	(8/4)	(12/6)	(15/8)	0.994	1.000	1.000	1.000
Location of stroke lesion ☨ (supratentorial/infratentorial)	(7/4)	(15/3)	(21/2)	0.136	0.6828	0.211	0.444
Laterality of stroke lesion (right/left)	(3/6)	(9/7)	(8/13)	0.430	0.9927	1.000	0.9927
Modified Rankin Scale on admission	4.3 ± 0.5 (4–5)	4.4 ± 0.5 (4–5)	4.8 ± 0.4 (4–5)	0.005	1.000	0.016	0.044
Modified Rankin Scale at discharge	4.2 ± 0.8 (2–5)	4.3 ± 0.5 (4–5)	4.8 ± 0.4 (4–5)	0.002	1.000	0.011	0.007
Admission dates (days)	124.9 ± 57.7 (16–207)	305.1 ± 205.3 (62–906)	299.3 ± 274.0 (43–1010)	0.047	0.032	0.191	1.000
Initiation timing of rehabilitation therapy from the onset of stroke (days)	547.2 ± 1109.0 (43–3383)	86.6 ± 40.5 (35–187)	122.6 ± 135.9 (34–593)	0.058	0.071	0.085	0.070
Period of rehabilitation therapy (days)	119.9 ± 57.1 (13–192)	298.4 ± 204.8 (56–898)	273.6 ± 275.6 (29–1004)	0.079	0.102	0.166	1.000
Motor subtotal FIM score on admission	31.7 ± 16.0 (13–55)	24.9 ± 8.5 (17–43)	16.7 ± 8.1 (13–42)	<0.001	1.000	0.002	<0.001
Cognitive subtotal FIM score on admission	16.0 ± 8.3 (5–30)	14.4 ± 5.6 (7–26)	9.6 ± 5.1 (5–26)	0.007	1.000	0.014	0.046
Total FIM score on admission	47.7 ± 23.9 (18–85)	39.3 ± 10.3 (27–59)	26.3 ± 11.1 (18–58)	<0.001	1.000	0.007	0.002
Motor subtotal FIM score at discharge	37.8 ± 16.0 (13–87)	27.7 ± 11.9 (16–53)	16.5 ± 8.3 (13–42)	<0.001	1.000	0.004	<0.001
Cognitive subtotal FIM score at discharge	17.2 ± 8.8 (5–32)	15.7 ± 5.5 (7–24)	9.6 ± 5.3 (5–26)	0.001	1.000	0.004	0.011
Total FIM score at discharge	54.9 ± 33.1 (18–119)	43.4 ± 14.5 (23–77)	26.0 ± 11.4 (18–58)	<0.001	1.000	0.004	<0.001
Chronological change of total FIM score	8.1 ± 14.7 (−18–44)	4.1 ± 7.5 (−4–19)	−0.3 ± 2.2 (−7–4)	0.019	0.569	0.016	0.329
Transfer FIM score on admission (walk/wheelchair/walk and wheelchair)	1.8 ± 1.5 (1–5)	1.2 ± 0.7 (1–4)	1.1 ± 0.4 (1–3)	0.113	0.347	0.121	1.000
Transfer FIM score at discharge (walk/wheelchair/walk and wheelchair)	3.3 ± 2.5 (1–6)	1.2 ± 0.7 (1–4)	1.1 ± 0.4 (1–3)	<0.001	<0.001	<0.001	1.000
BI score on admission	30.8 ± 25.7 (0–85)	26.1 ± 18.3 (0–55)	8.5 ± 15.8 (0–55)	0.002	1.000	0.008	0.001
BI score at discharge	47.5 ± 36.9 (0–100)	29.7 ± 20.5 (5–30)	7.6 ± 16.3 (0–55)	<0.001	0.147	<0.001	0.013
Chronological change of BI score	16.7 ± 16.4 (−5–45)	3.6 ± 11.7 (−20–25)	−0.9 ± 7.5 (−30–10)	<0.001	0.101	0.001	0.474

Mean ± standard deviation (minimum score—maximum score); Home: patients discharged home, Geriatric Facilities: patients discharged to geriatric facilities, Another hospital: patients transferred to another hospital; BI: Barthel index, FIM: functional independence measure Kruskal–Wallis test (post hoc analysis: Bonferroni correction), *: one-way analysis of variance (post hoc analysis: Bonferroni correction), ☨: Fisher’s Exact Test (post hoc analysis: Holm correction).

## Data Availability

Not applicable.
